# Supplementary material for: FAM50A as a novel prognostic marker modulates the proliferation of colorectal cancer cells via CylinA2/CDK2 pathway
Source: PLoS One. 2025 Feb 25;20(2):e0318776. doi: 10.1371/journal.pone.0318776 (PMC11856281; doi:10.1371/journal.pone.0318776)
Supplement: S1 File — (ZIP) [file pone.0318776.s001.zip › raw.data/S1_raw_images.pdf]

Fig 6

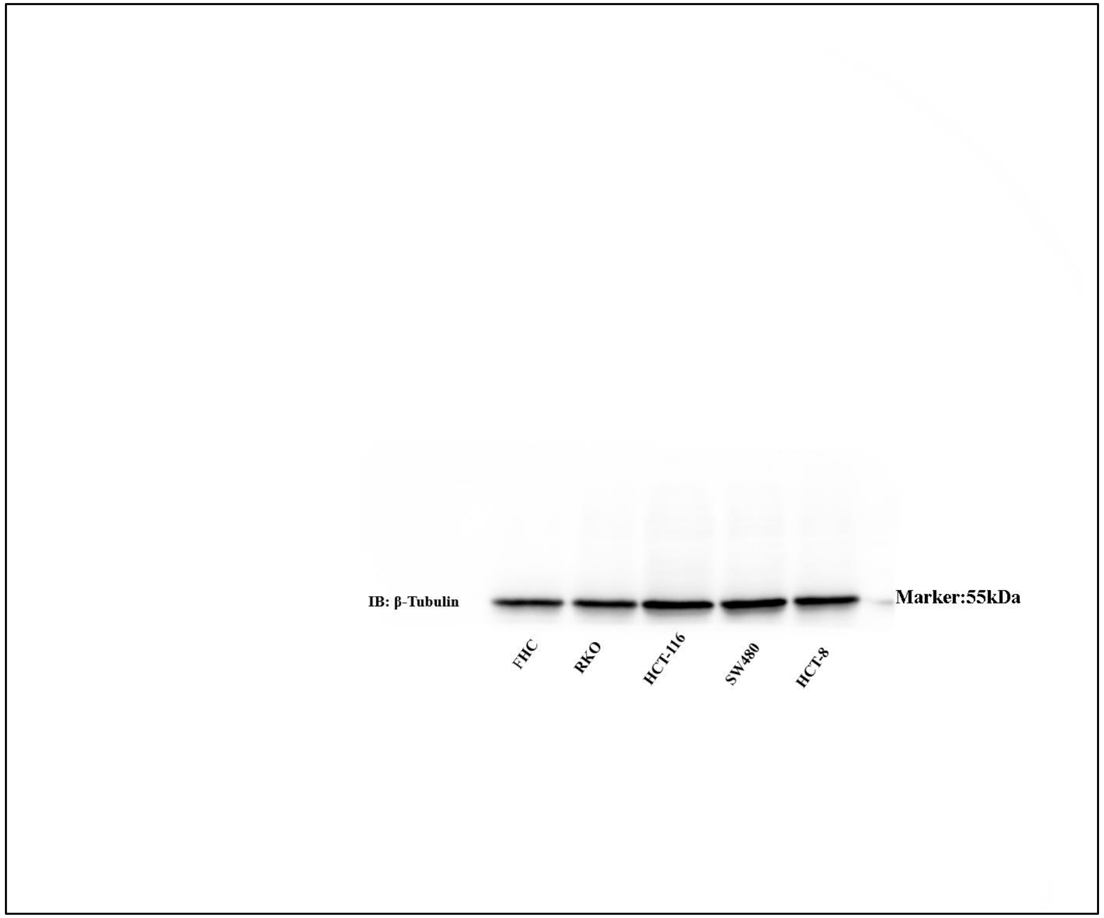

IB: β-Tubulin
Marker:55kDa
FHC
RKO
HCT-116
SW480
HCT-8

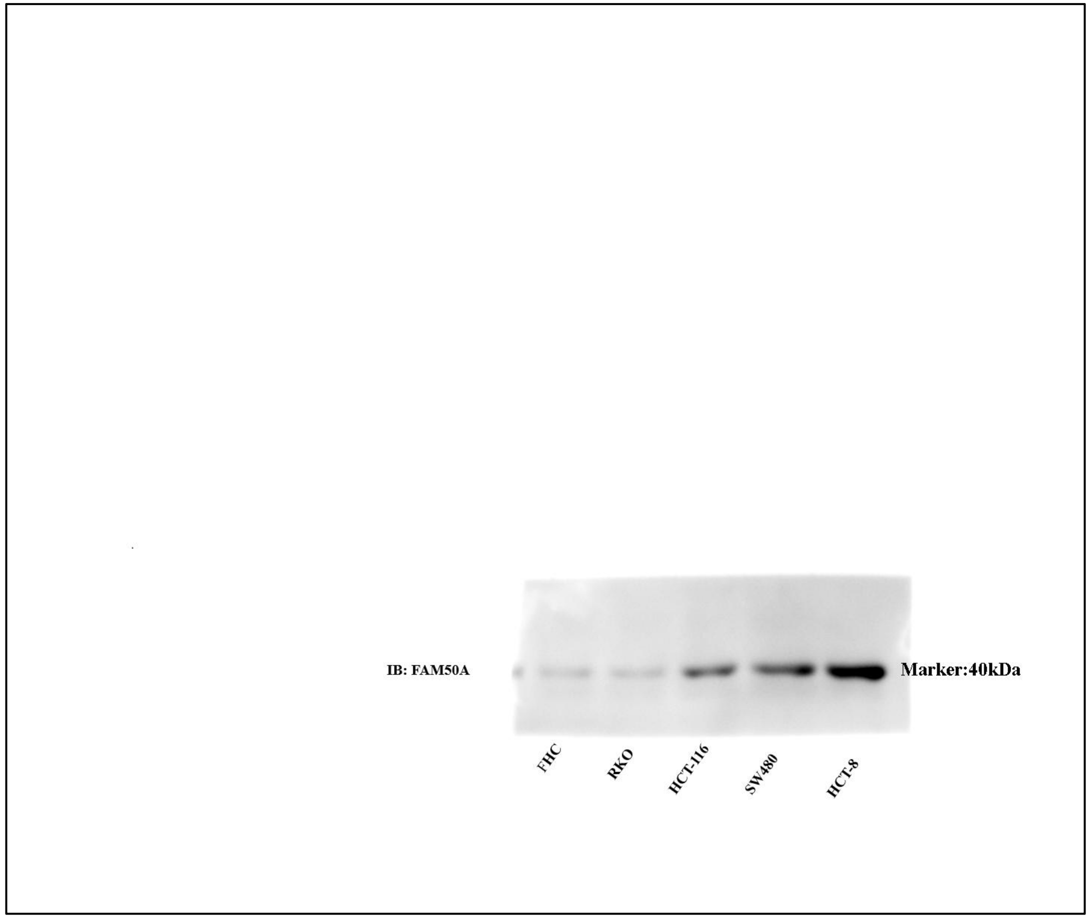

IB: FAM50A
Marker:40kDa
FHC
RKO
HCT-116
SW480
HCT-8

Fig 6

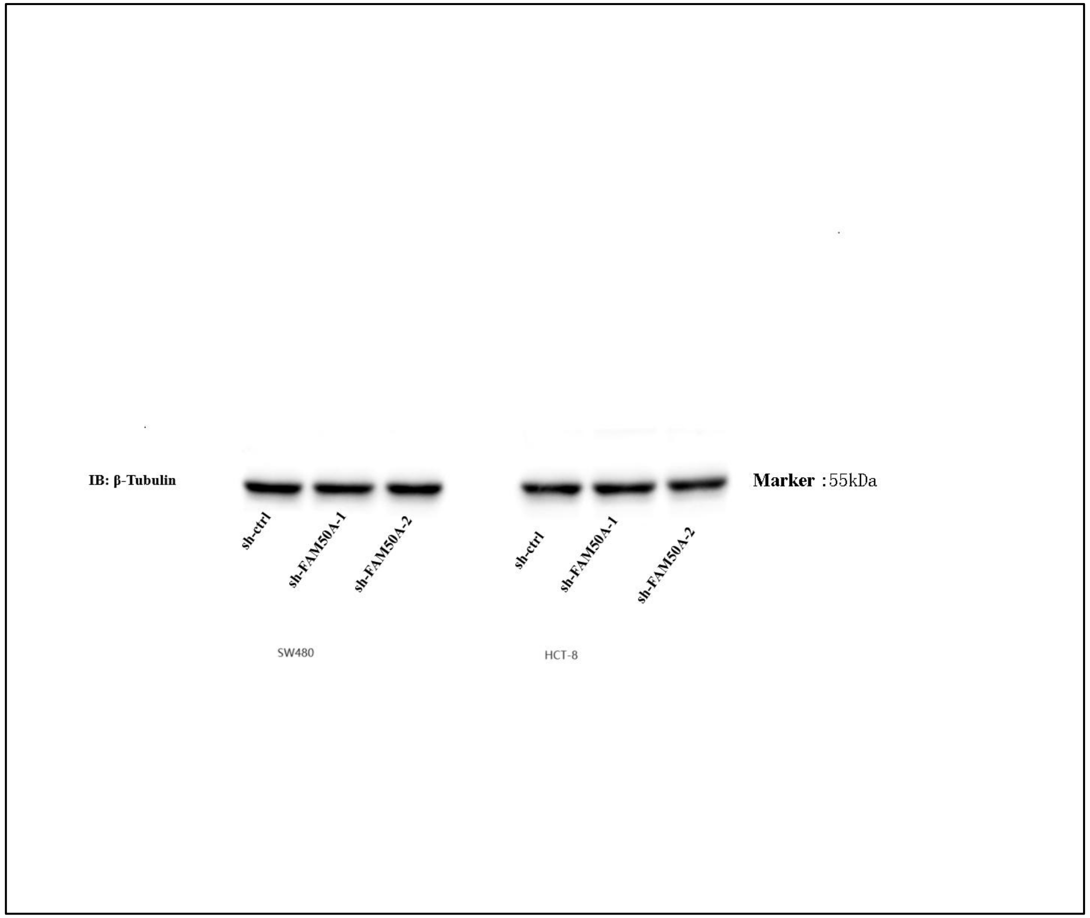
IB: β-Tubulin
Marker :55kDa
sh-ctrl
sh-FAM50A-1
sh-FAM50A-2
sh-ctrl
sh-FAM50A-1
sh-FAM50A-2
SW480
HCT-8

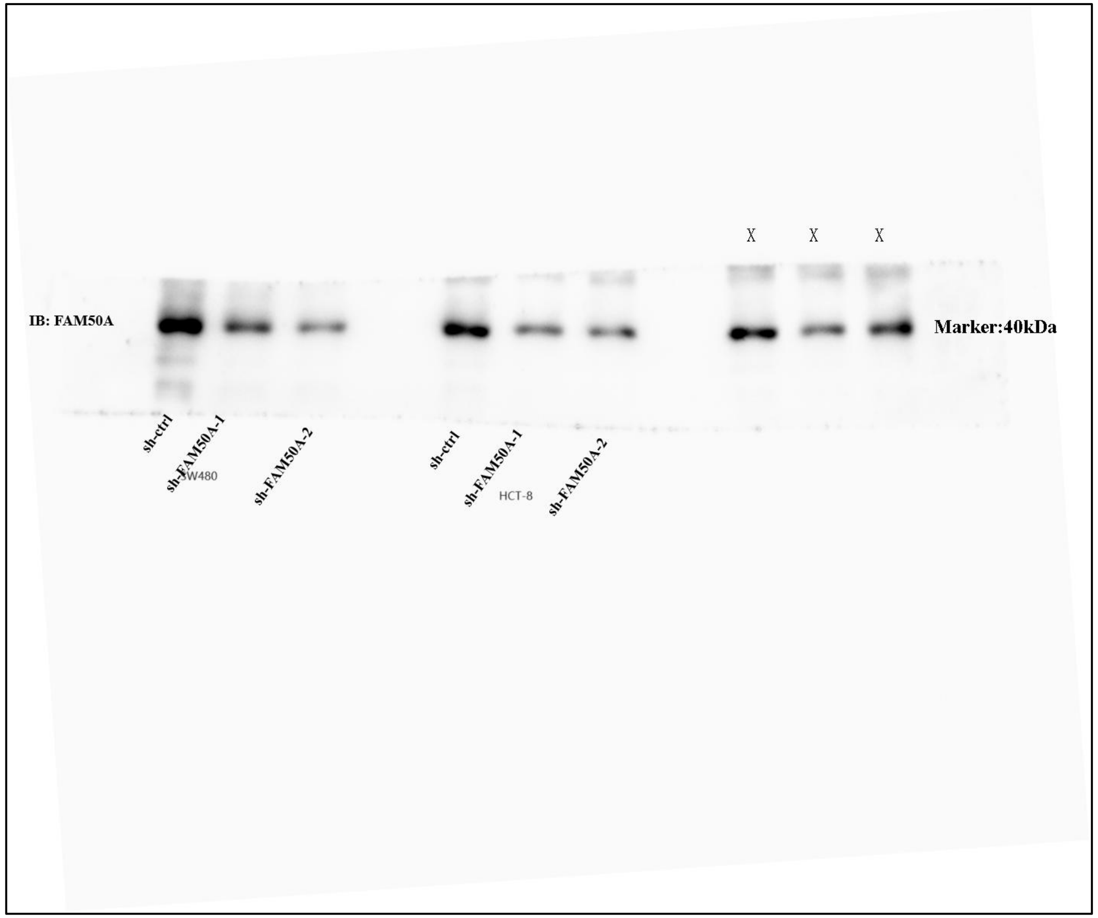
X
X
X
IB: FAM50A
Marker:40kDa
sh-ctrl
sh-FAM50A-1
SW480
sh-FAM50A-2
sh-ctrl
sh-FAM50A-1
HCT-8
sh-FAM50A-2

Fig 7

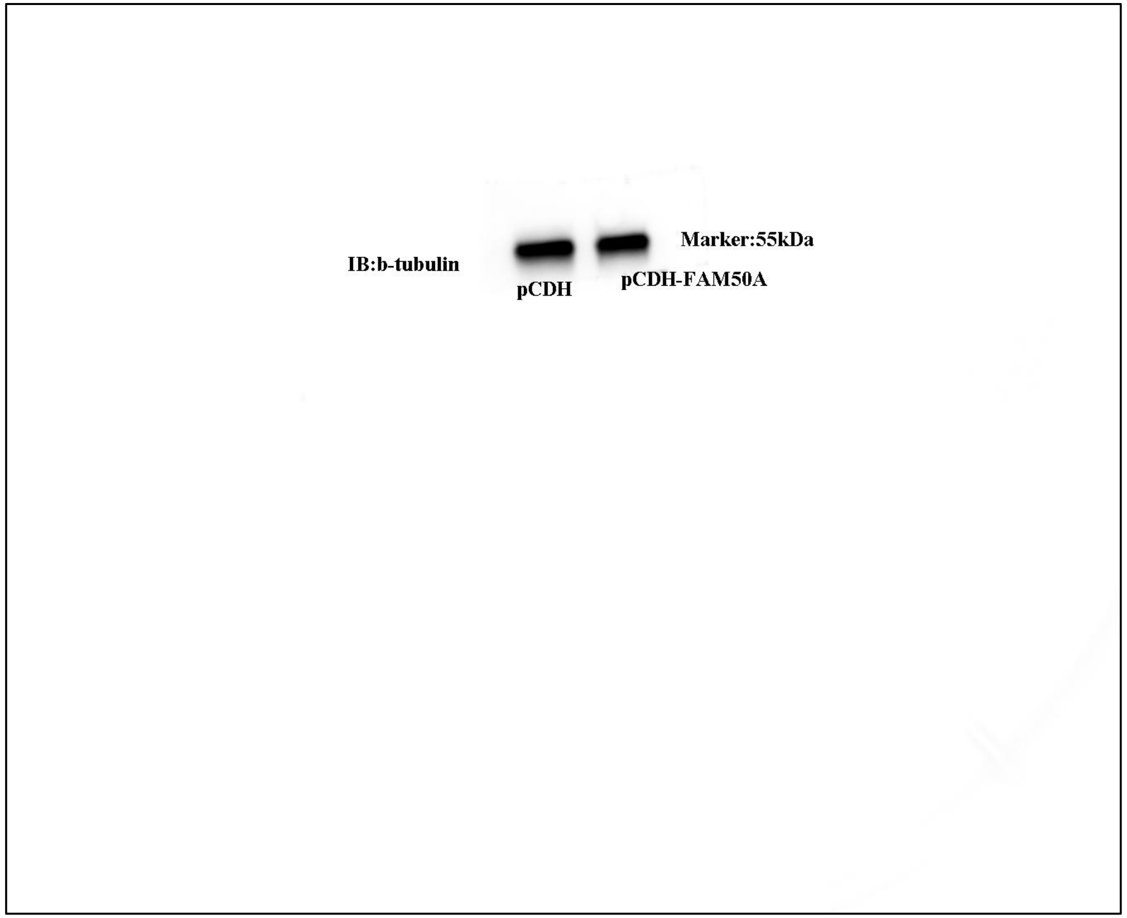
IB:b-tubulin
Marker:55kDa
pCDH
pCDH-FAM50A

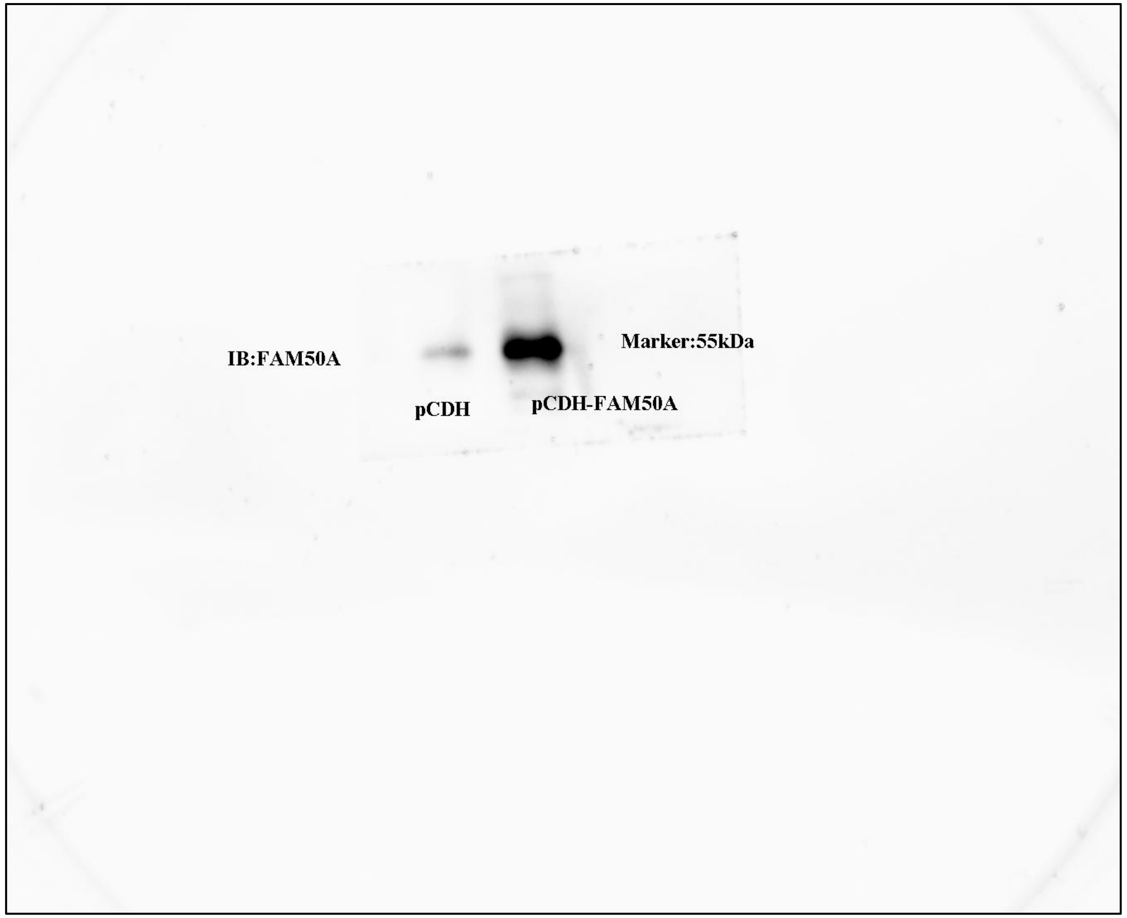
IB:FAM50A
Marker:55kDa
pCDH
pCDH-FAM50A

Fig 8 (in SW480)

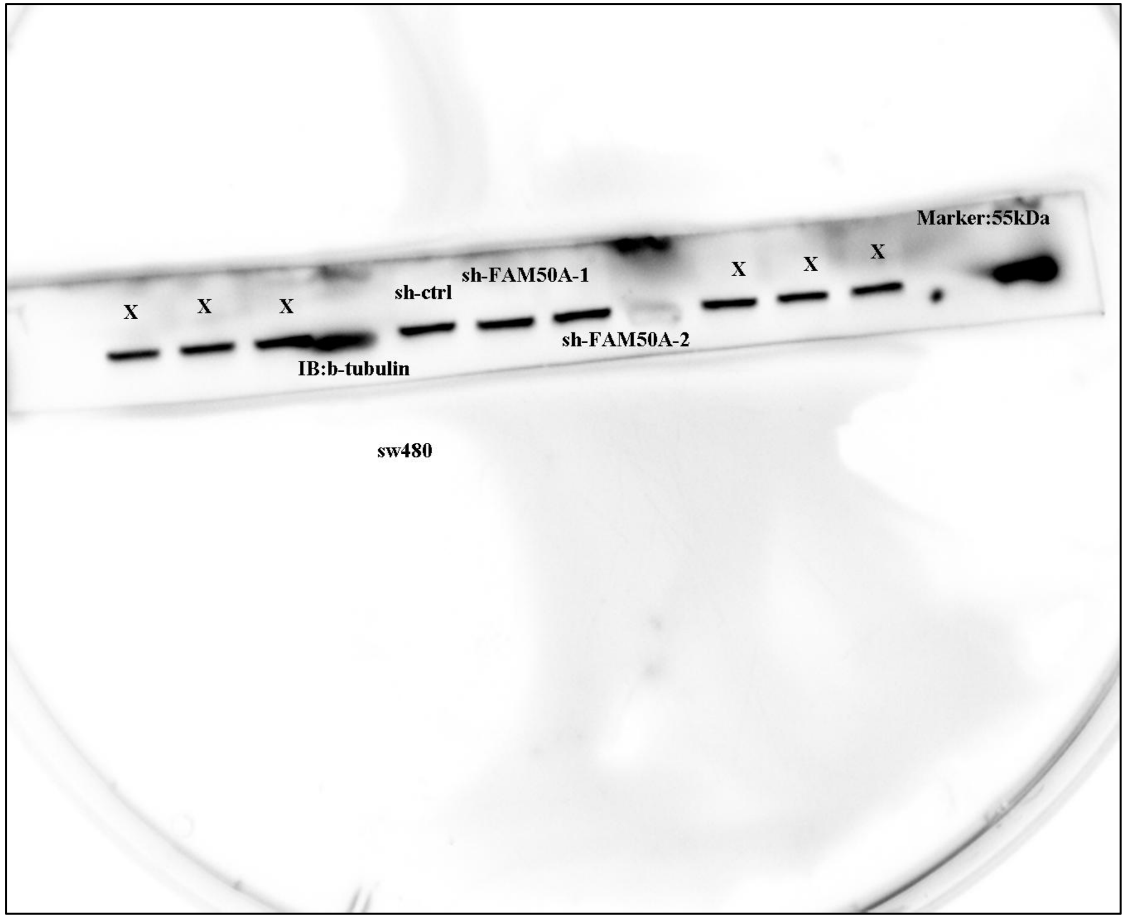

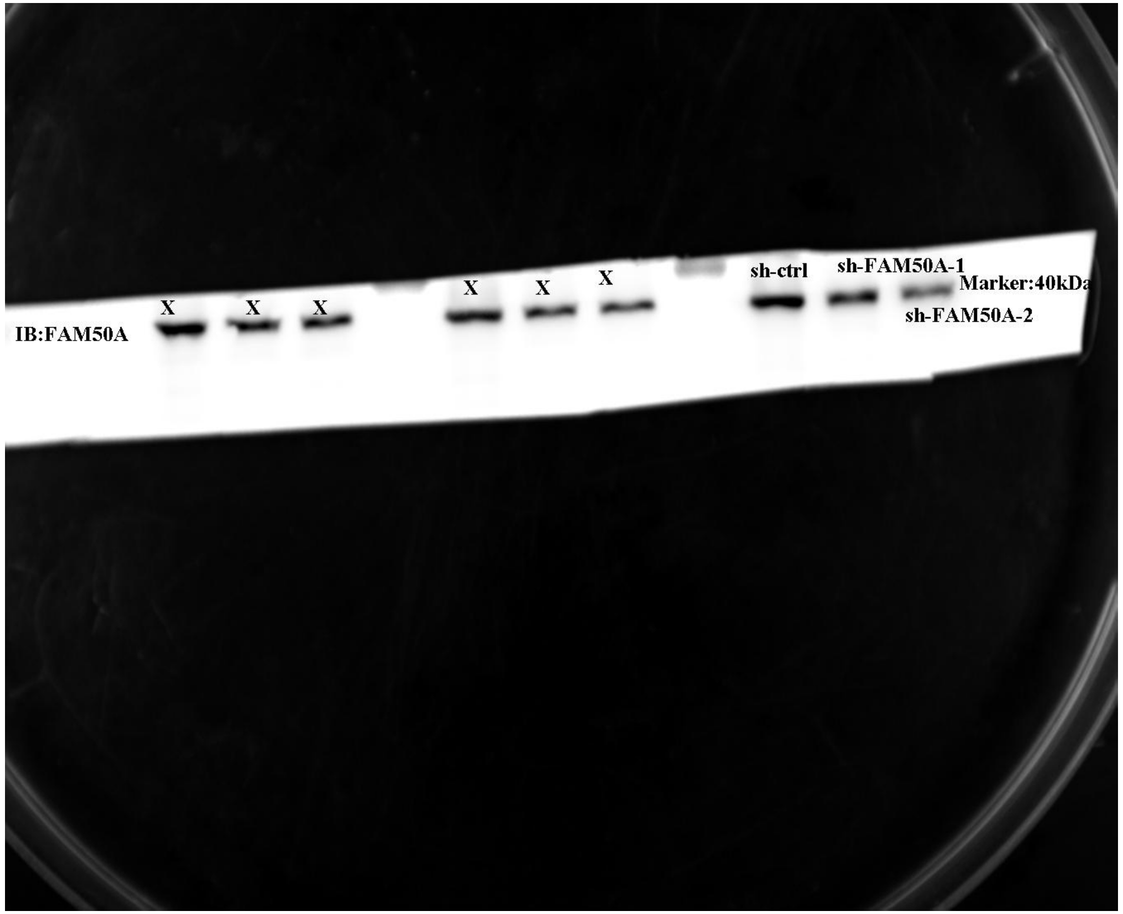

Fig 8 (in SW480)

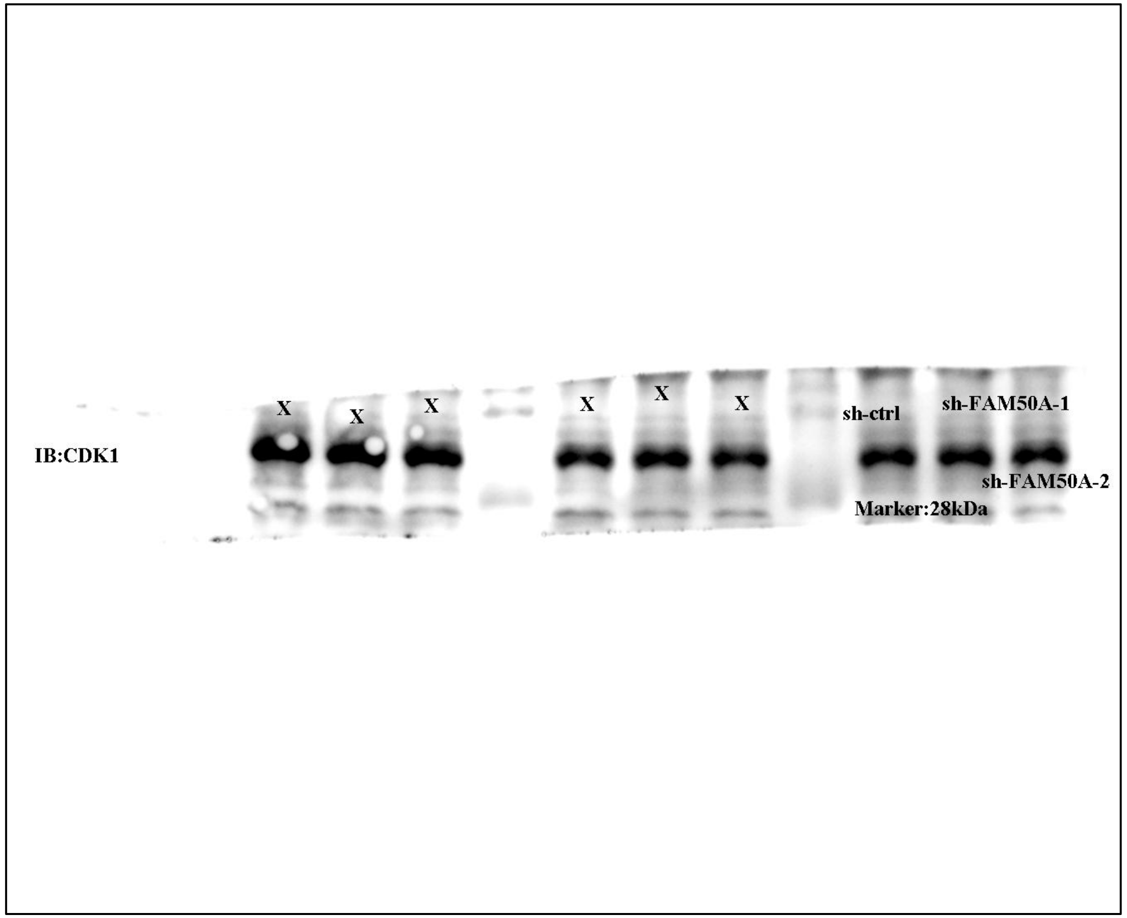

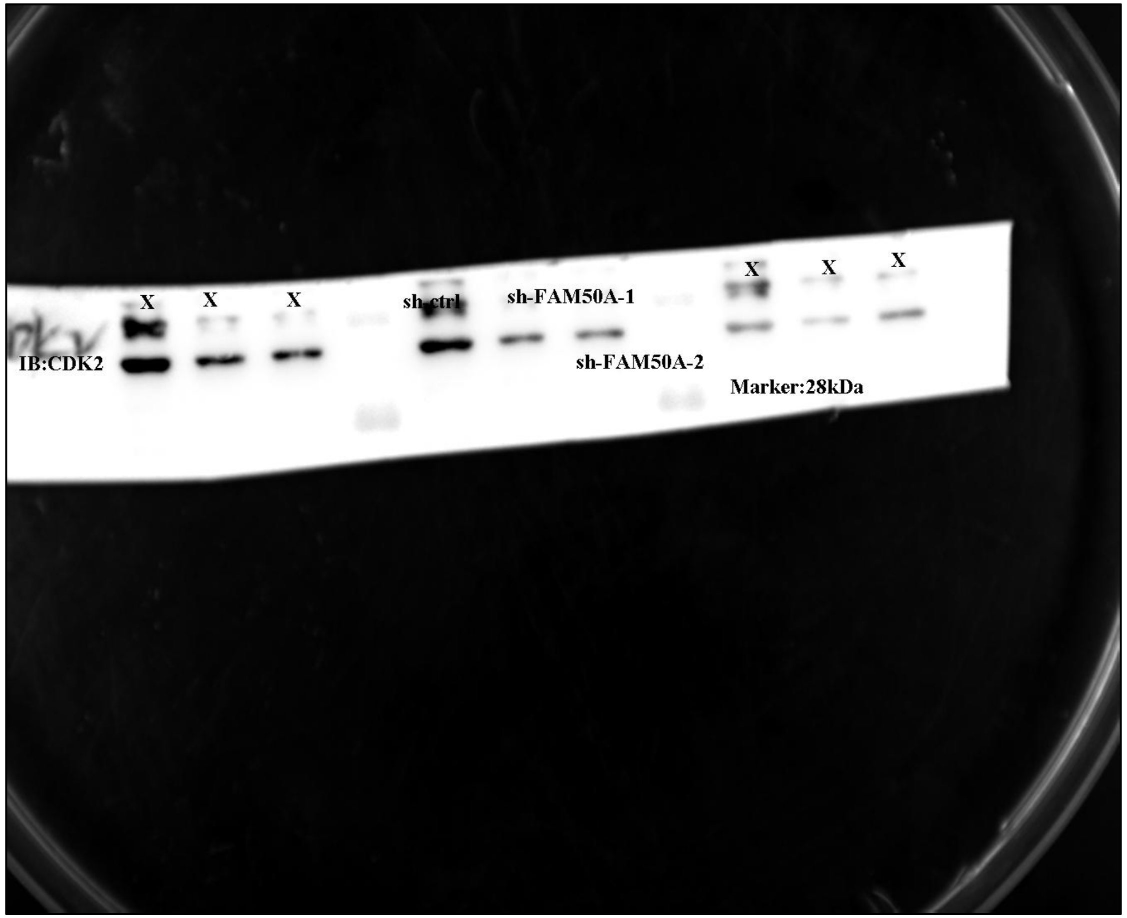

Fig 8 (in SW480)

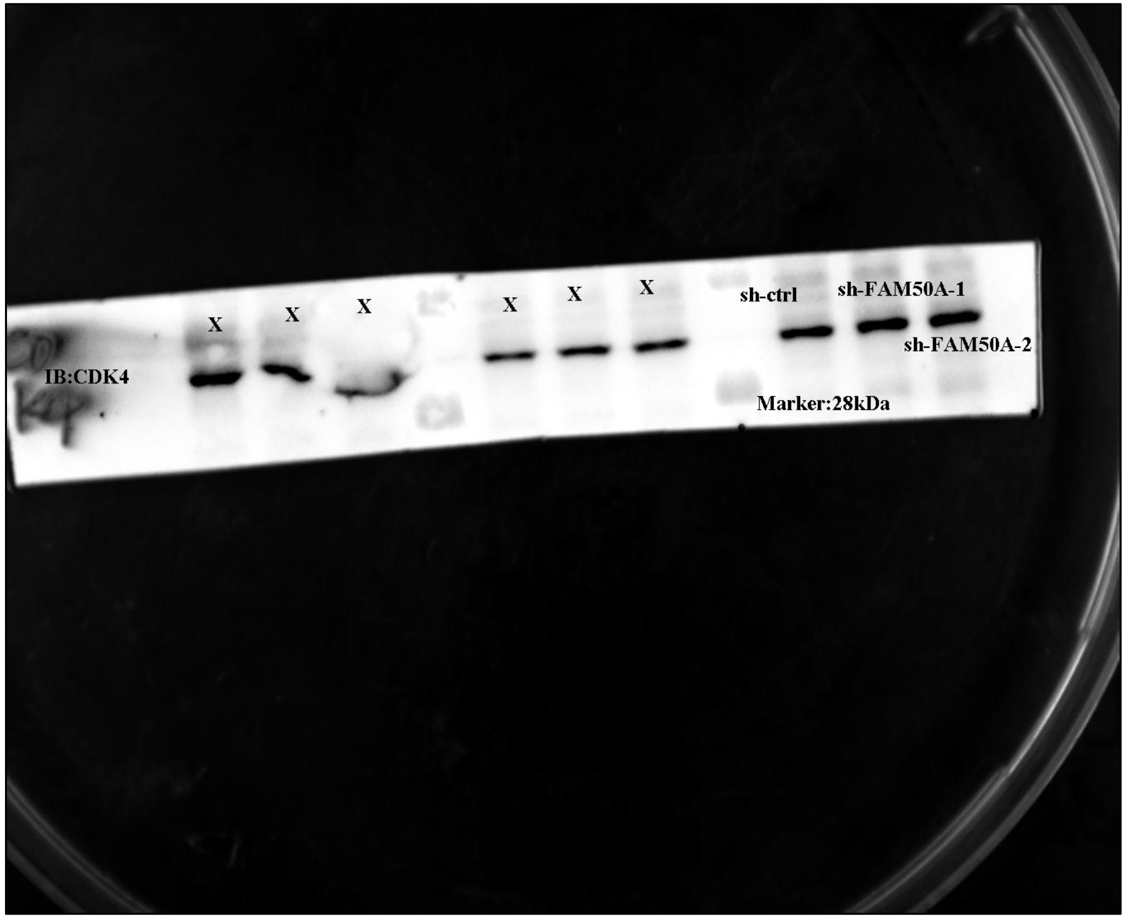

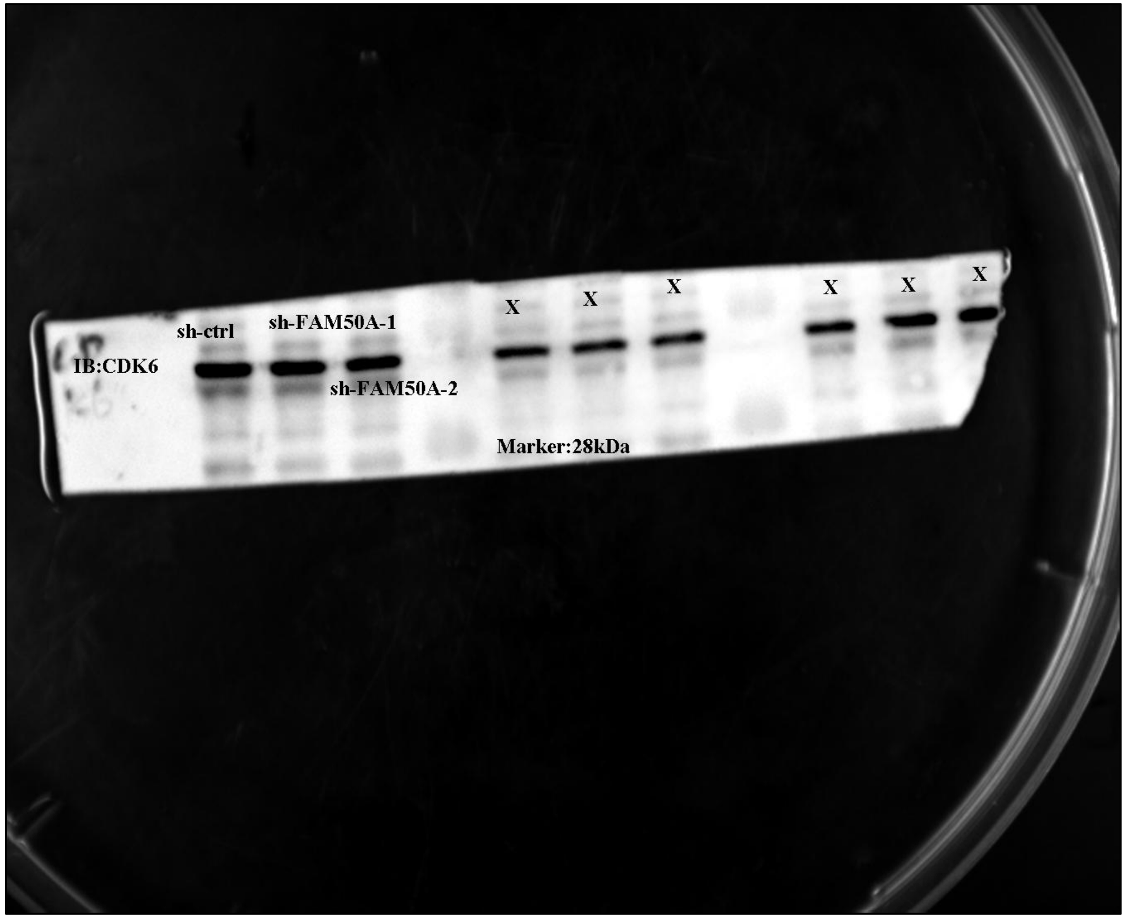

Fig 8 (in SW480)

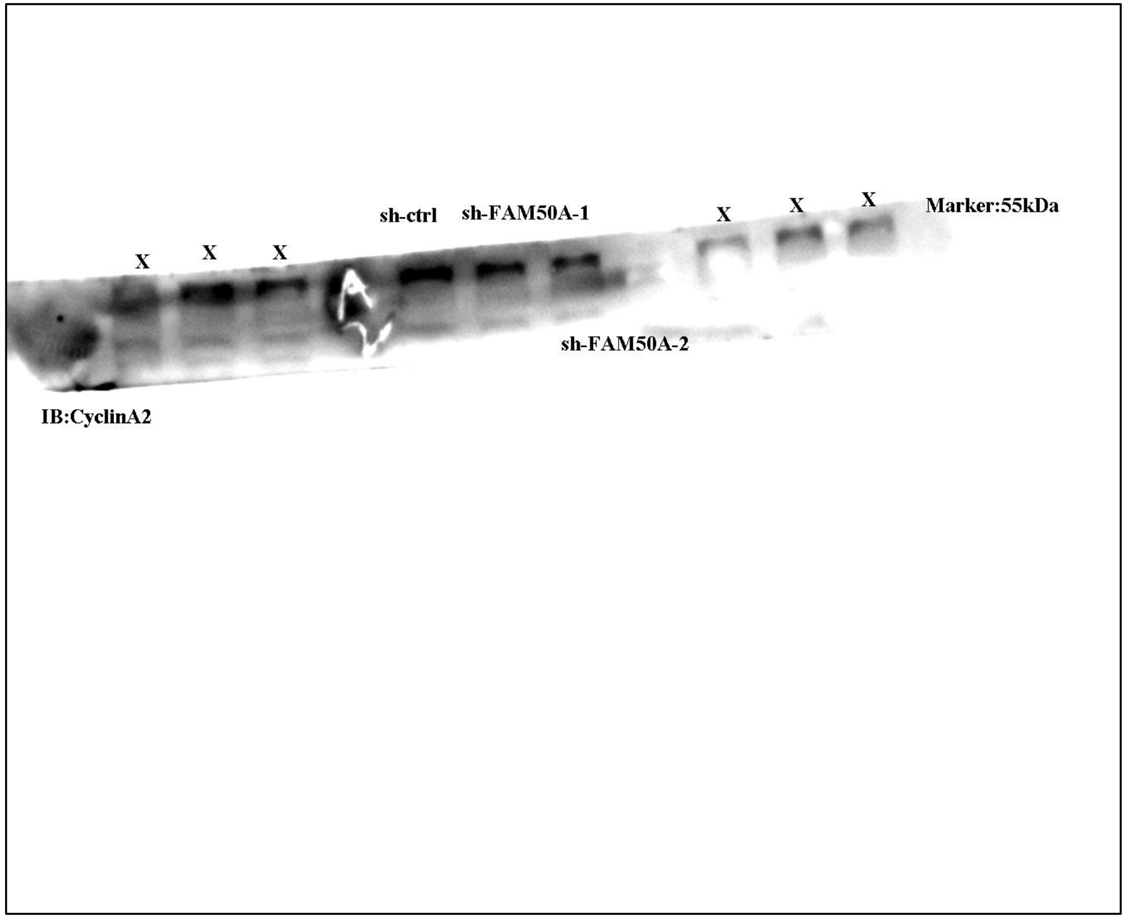

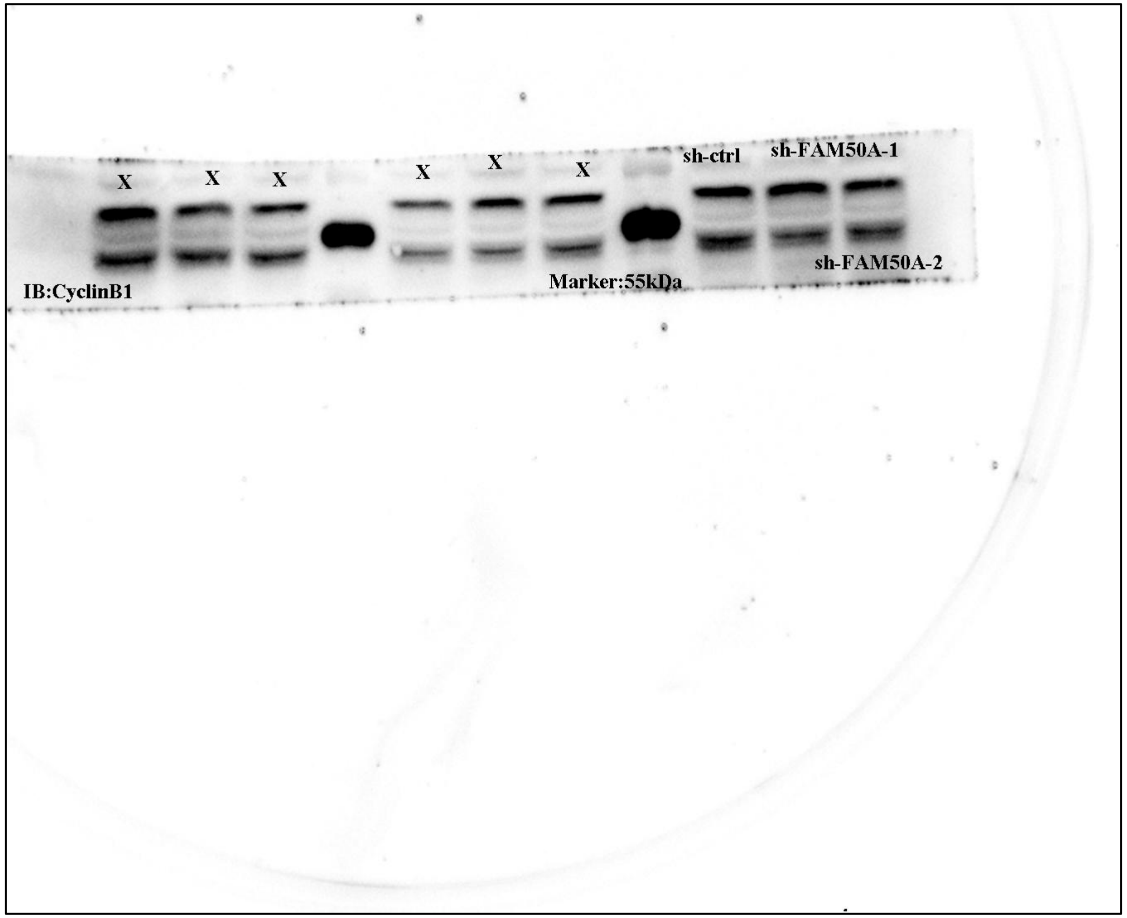

Fig 8 (in SW480)

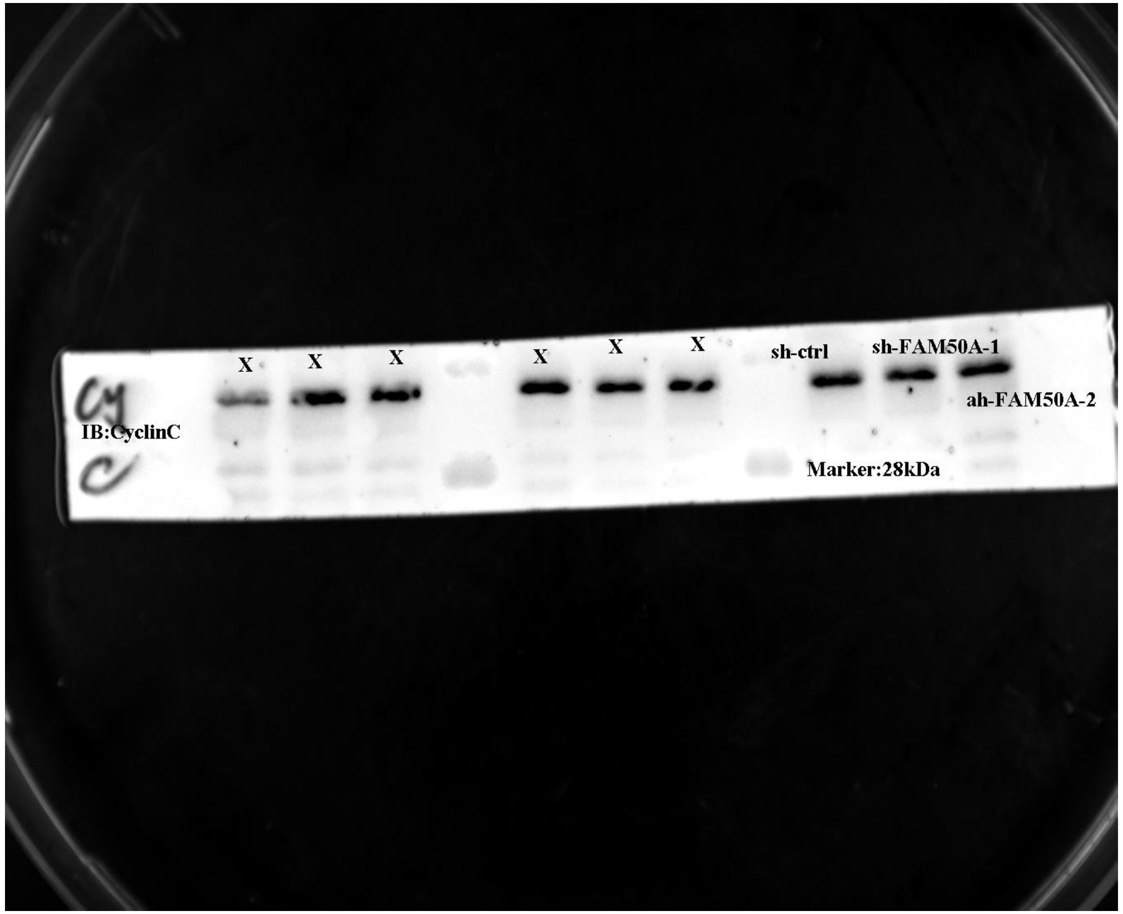

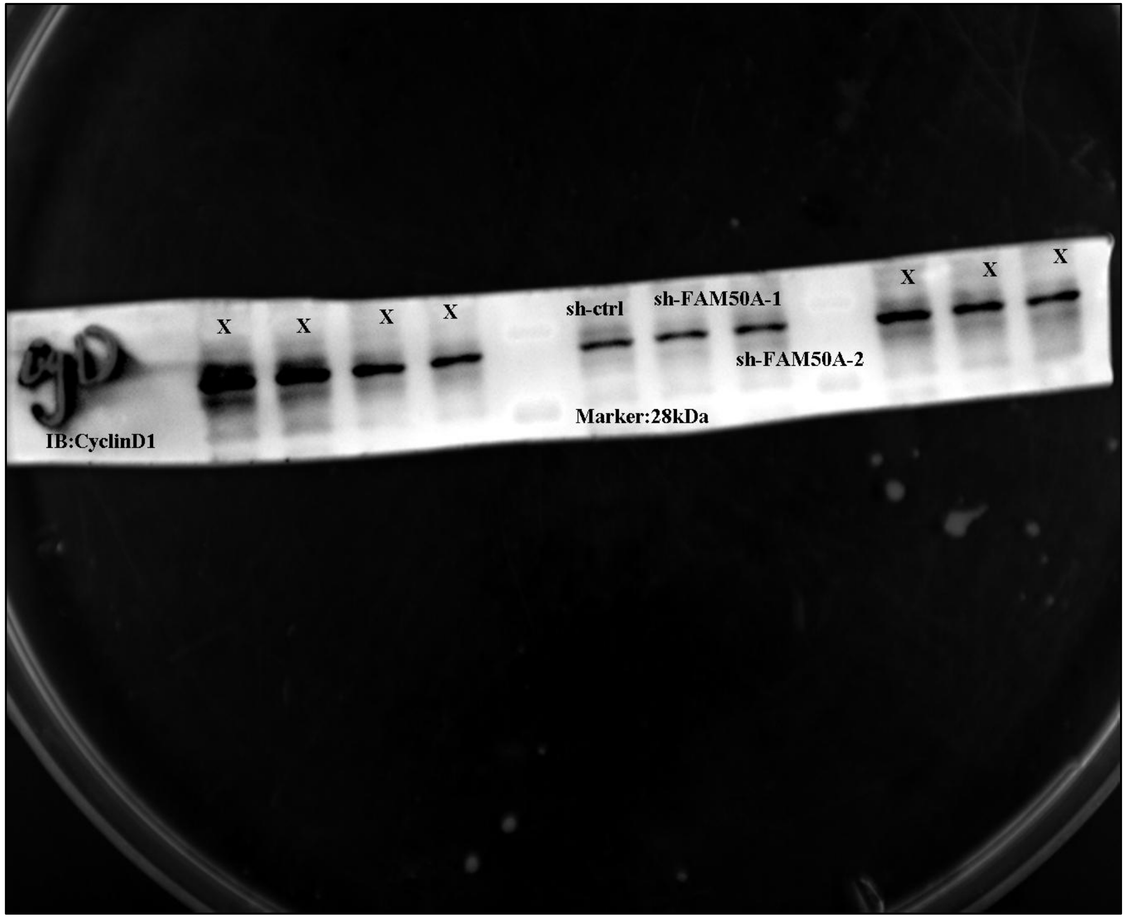

Fig 8 (in HCT-8)

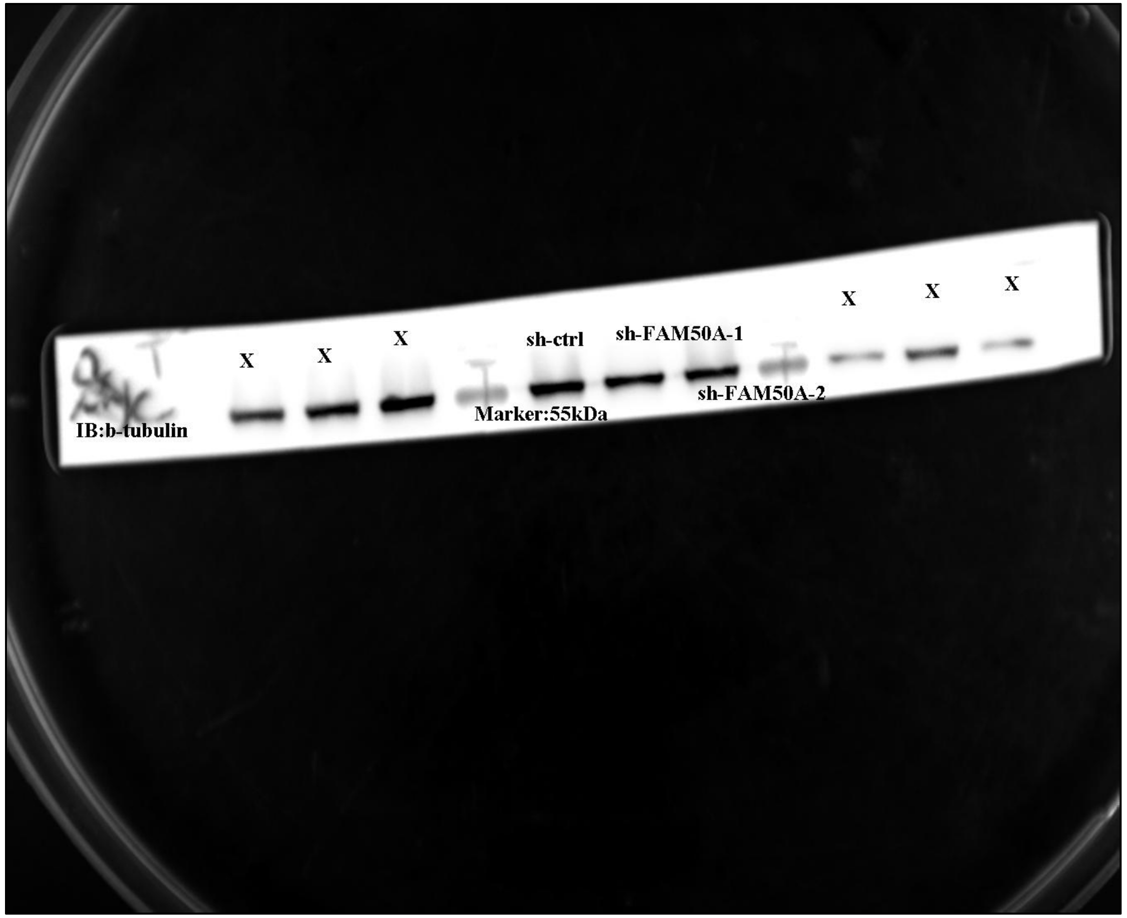
X
X
X
sh-ctrl
sh-FAM50A-1
X
X
X
sh-FAM50A-2
Marker:55kDa
IB:b-tubulin

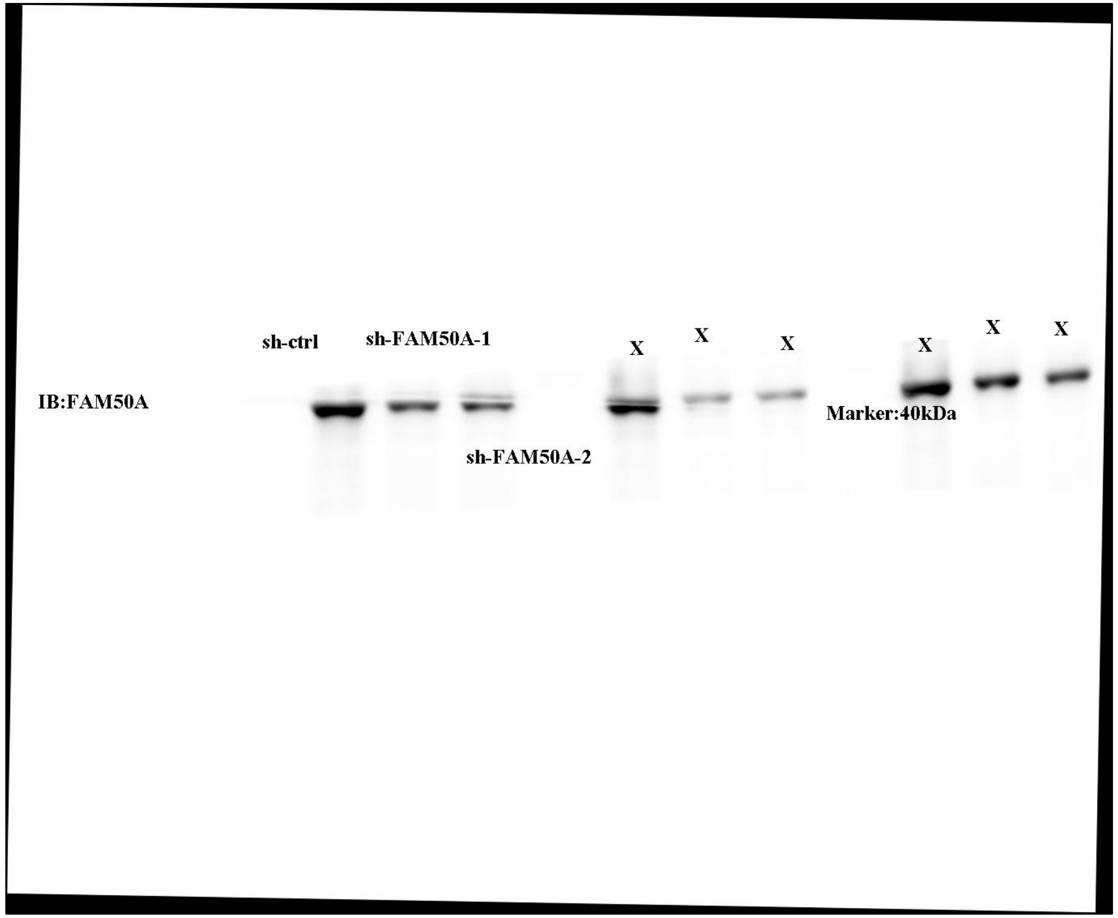
sh-ctrl
sh-FAM50A-1
X
X
X
X
X
X
IB:FAM50A
Marker:40kDa
sh-FAM50A-2

Fig 8 (in HCT-8)

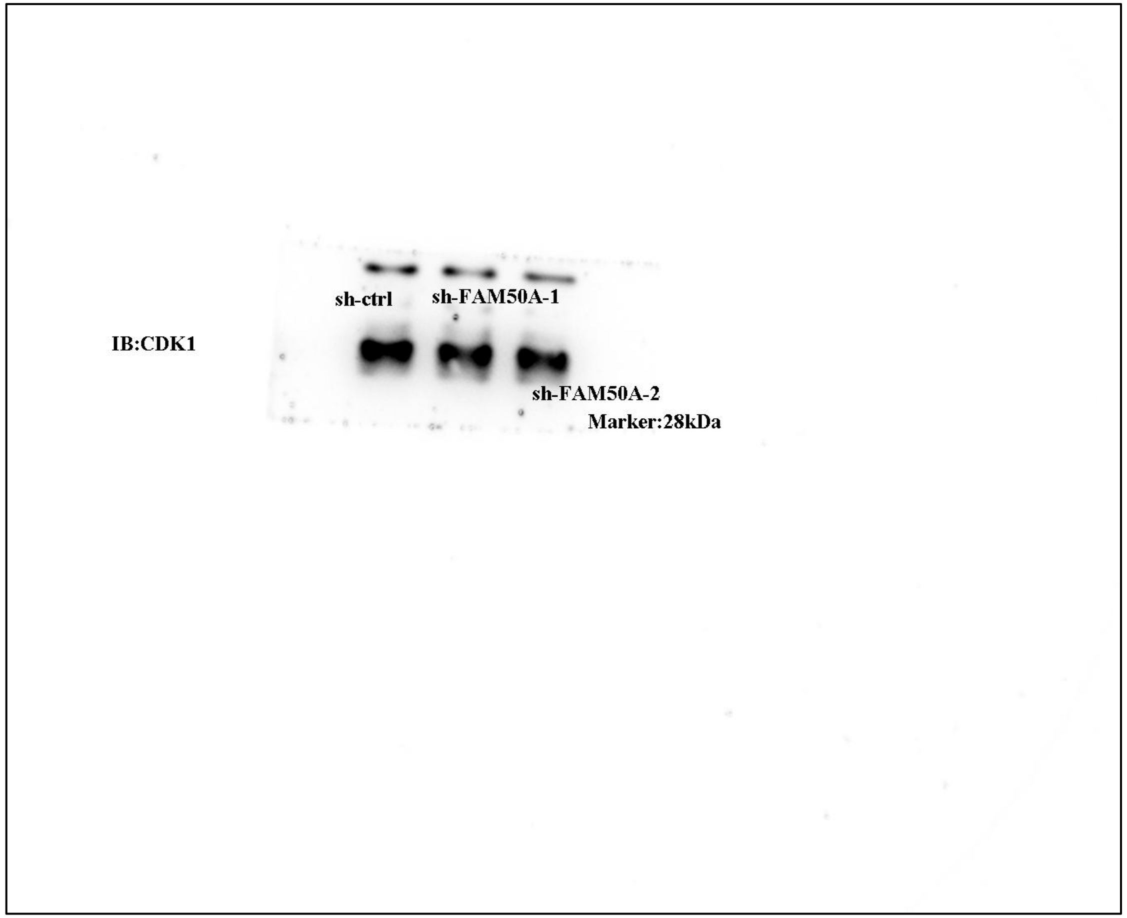
sh-ctrl
sh-FAM50A-1
IB:CDK1
sh-FAM50A-2
Marker:28kDa

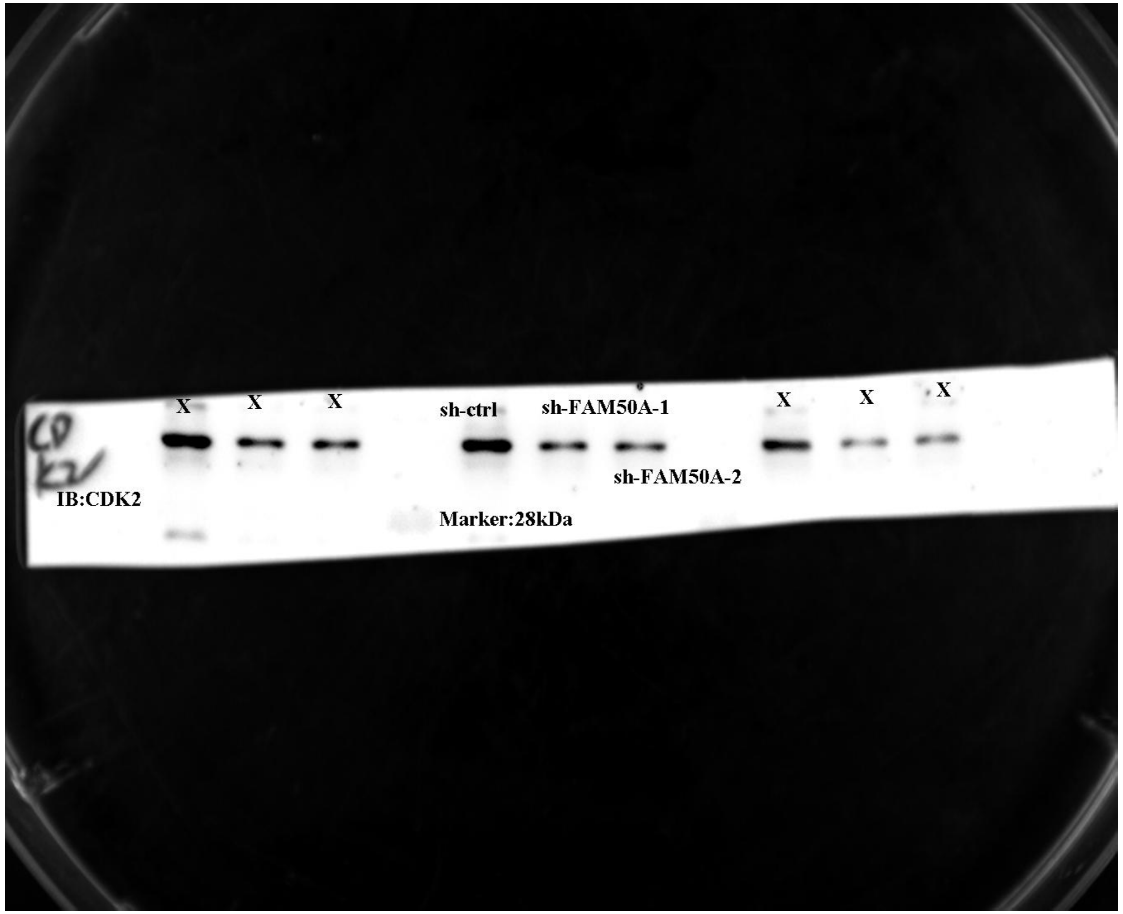
X
X
X
sh-ctrl
sh-FAM50A-1
X
X
X
IB:CDK2
sh-FAM50A-2
Marker:28kDa

Fig 8 (in HCT-8)

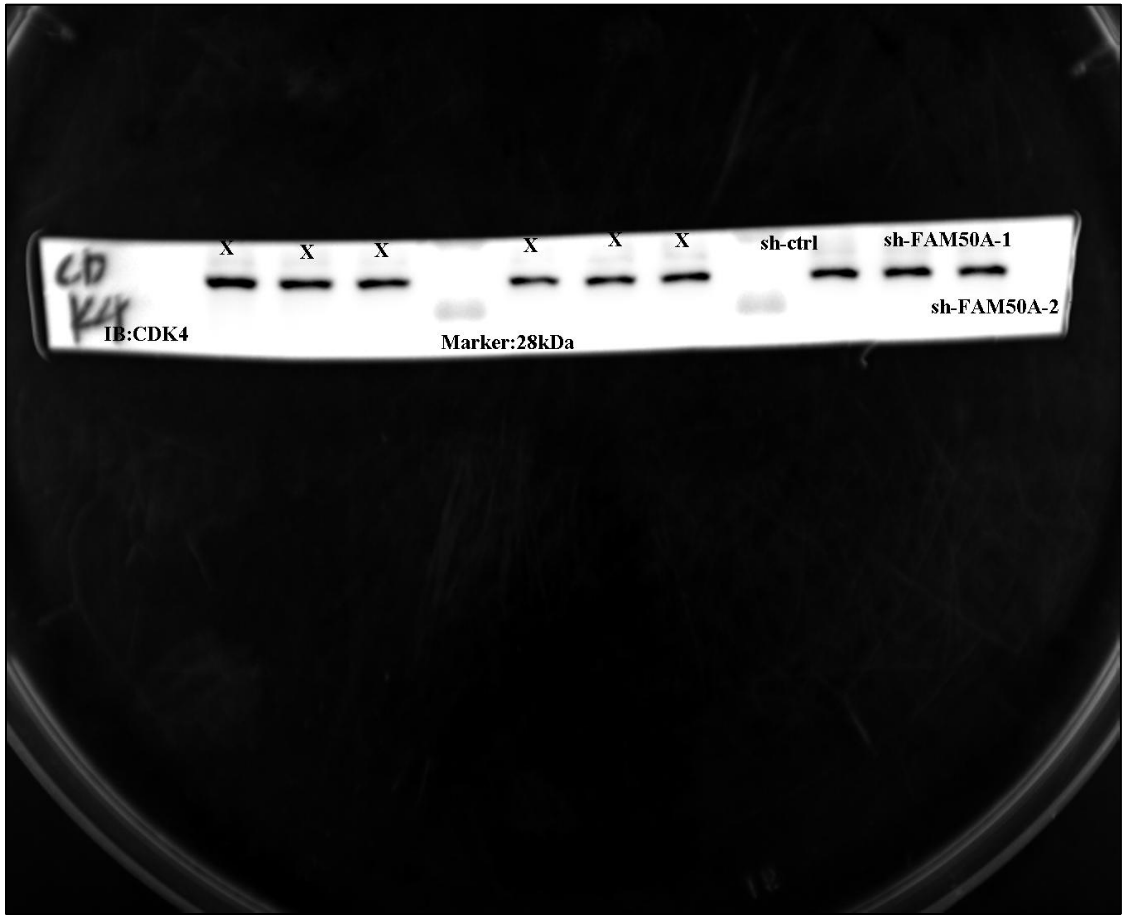
X
X
X
X
X
X
sh-ctrl
sh-FAM50A-1
sh-FAM50A-2
IB:CDK4
Marker:28kDa

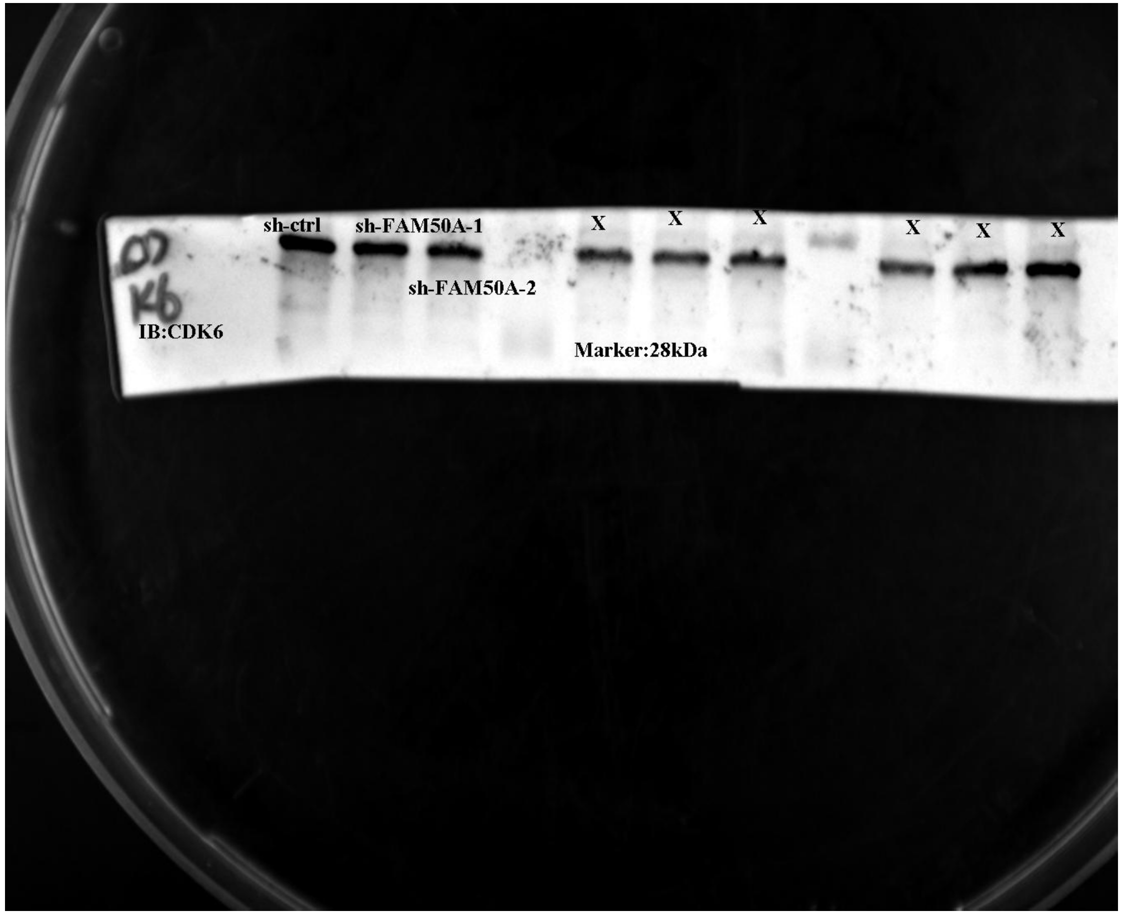
sh-ctrl
sh-FAM50A-1
sh-FAM50A-2
X
X
X
X
X
X
IB:CDK6
Marker:28kDa

Fig 8 (in HCT-8)

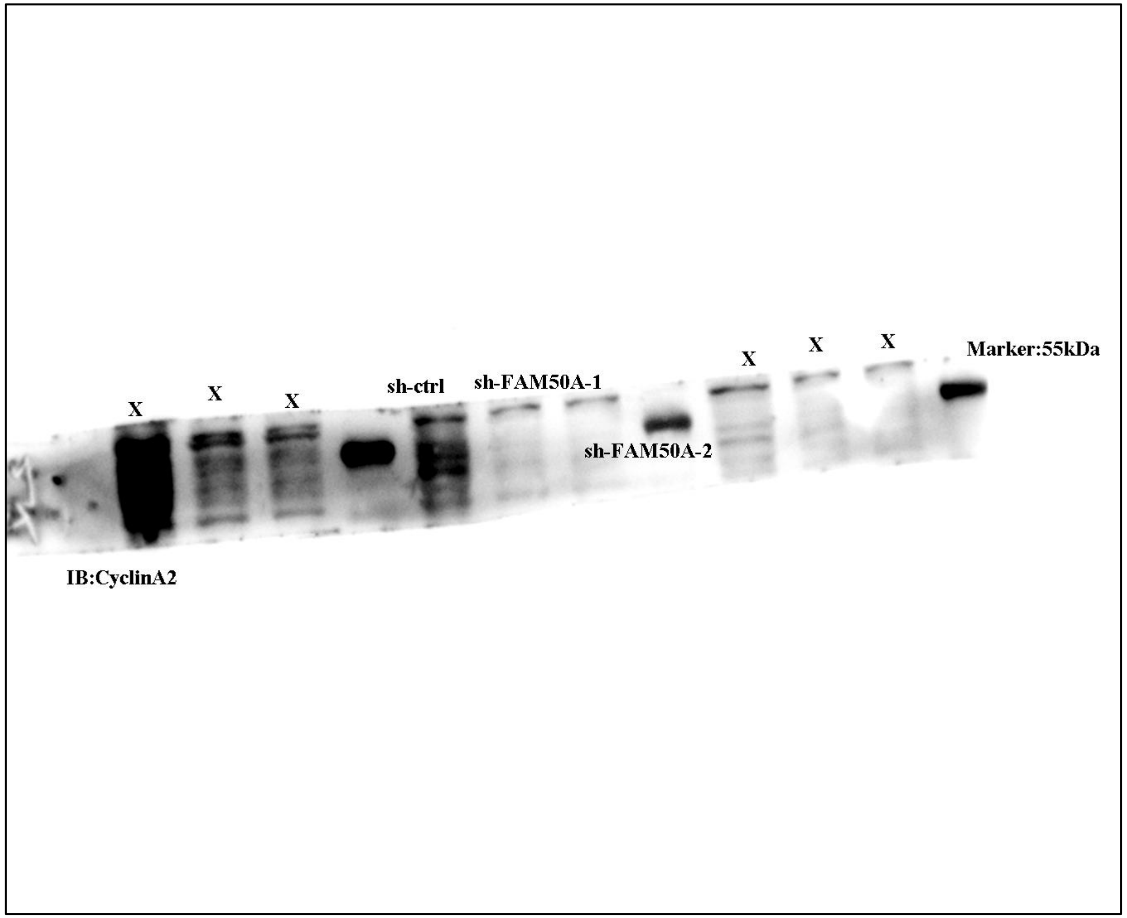
X
X
X
sh-ctrl
sh-FAM50A-1
sh-FAM50A-2
X
X
X
Marker:55kDa
IB:CyclinA2

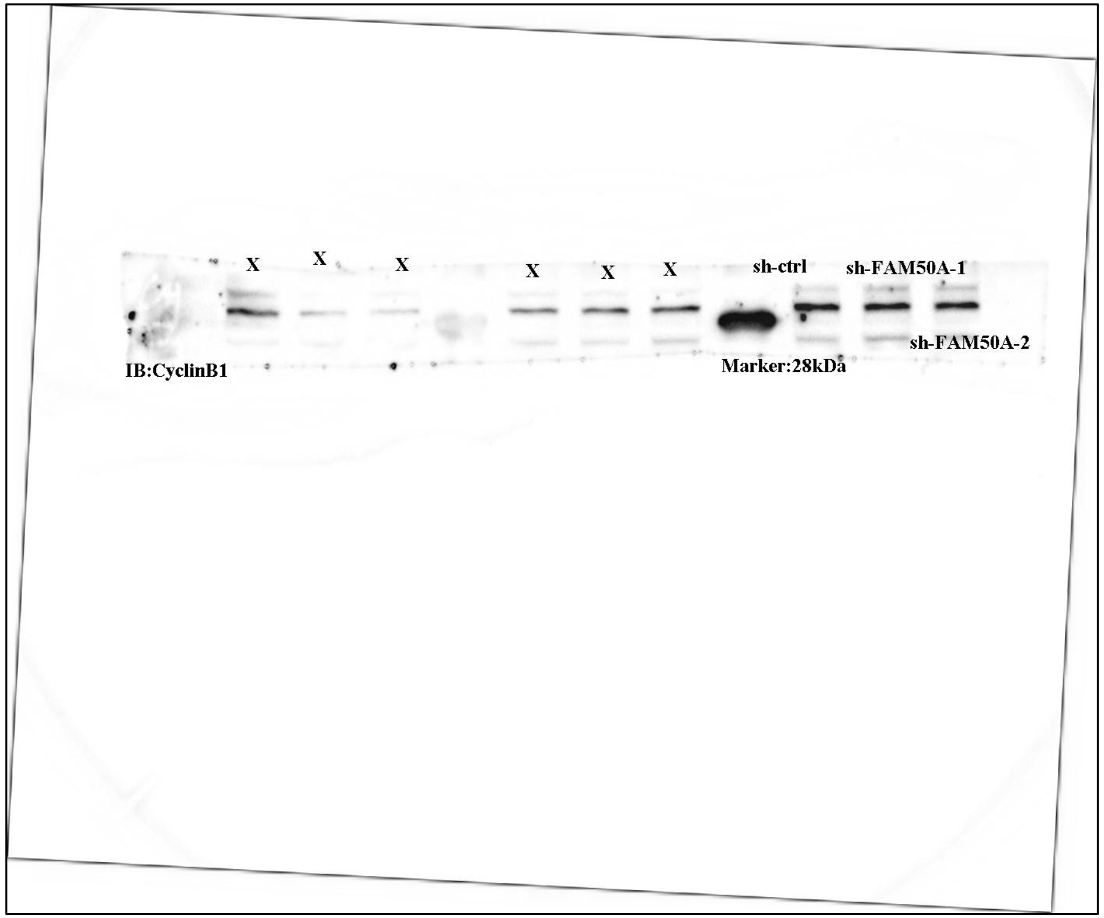
X
X
X
X
X
X
sh-ctrl
sh-FAM50A-1
sh-FAM50A-2
Marker:28kDa
IB:CyclinB1

Fig 8 (in HCT-8)

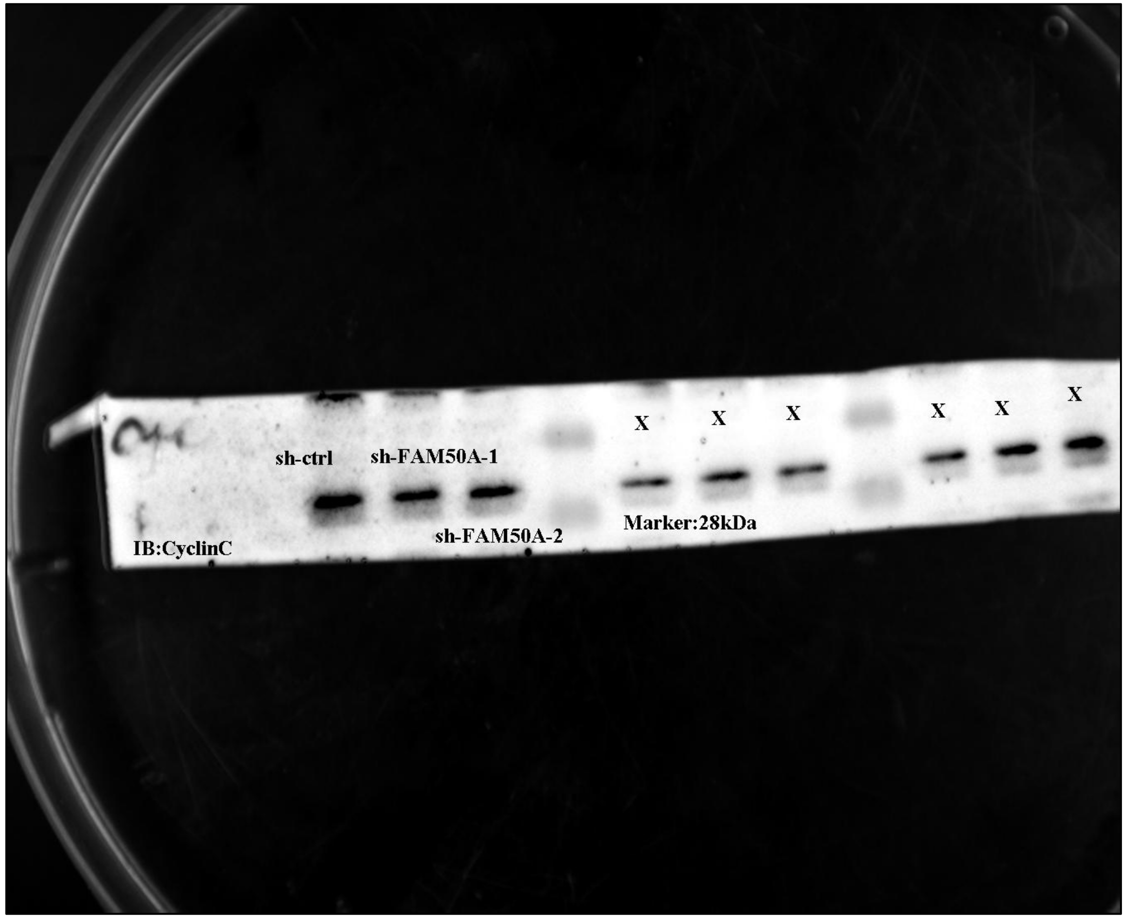
sh-ctrl
sh-FAM50A-1
sh-FAM50A-2
Marker:28kDa
IB:CyclinC
X
X
X
X
X
X

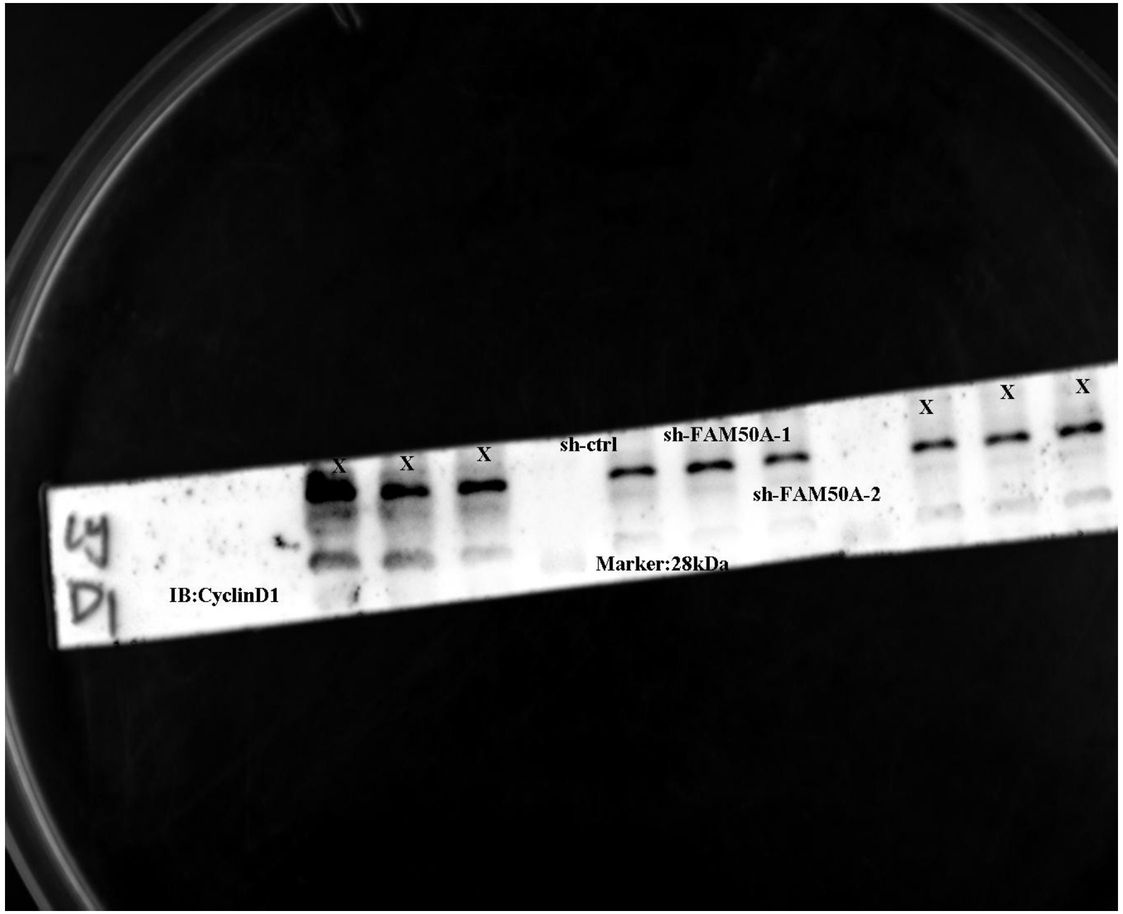
X
X
X
sh-ctrl
sh-FAM50A-1
sh-FAM50A-2
Marker:28kDa
IB:CyclinD1
X
X
X
