# Supplementary material for: FAM50A as a novel prognostic marker modulates the proliferation of colorectal cancer cells via CylinA2/CDK2 pathway
Source: PLoS One. 2025 Feb 25;20(2):e0318776. doi: 10.1371/journal.pone.0318776 (PMC11856281; doi:10.1371/journal.pone.0318776)
Supplement: Attachment — Submitted filename: Response to Reviewers.doc [file pone.0318776.s002.doc]

To editor:

We have re-entered the original data into the table as the "minimal data set"and uploaded the table as a file in Supporting information item. Please find it in system.

To reviewers:

Reviewer #1: This study identified FAM50A as a potential novel prognostic marker for colorectal cancer (CRC) and, for the first time, clarified its role in regulating CRC cell proliferation via the CyclinA2/CDK2 pathway. This offers a new perspective and potential target for the diagnosis and treatment of CRC. This article suggests that FAM50A may promote tumor cell proliferation via the CyclinA2/CDK2 signaling pathway, the direct interaction for this mechanism needs to be improved, as least some interaction models.
Specific issues in the text should be clarified:
1.In the text, the 252nd line mentions ‘Using ROC curve analysis’This should be reflected in the figure with the inclusion of an ROC curve graph.

ANSWER: With reference to your valuable suggestions, we have recalculated the optimal cut-off value of the scores and drawn the ROC curve in Figure 2A, 2B. And we have also reorganized the layout of Figure 2.
2.In academic papers, it is crucial to provide clear and concise figure legends to assist readers in quickly comprehending the content of the figures. The subplot labels ABCD in Figure 6 and 7 are inconsistent with other figure formats and should be standardized.

ANSWER: We have already made the revisions.

3.The figure 6 caption has a mismatch between parts F and G, and it is suggested that a statistical analysis should be conducted on the 'Edu' part to make it more concise.

ANSWER: We have already added the statistical description of the EdU results and adjusted the positions of F and G at the same time.

4.It is suggested that a flowchart be added to Figures A and B in Figure 8 to enhance the persuasiveness of the evidence.

ANSWER: Referring to your suggestions, we have added the flow cytometry result images.
5.Clearly label the samples on the WB (Western blot) lanes to visually compare the detection results of each lane in Figure 8.

ANSWER: We have already added the labels for the samples of WB lane .

6. Figure 3 and Figure 6 F should include the "n" value in the statistical graphs to ensure the transparency, reproducibility, and reliability of the research results.

ANSWER: Thank you very much for your suggestions. With reference to your advice, we have revised the figures in the article as much as possible to show the "n" value in the statistical graphs.

Reviewer #2: I have reviewed the manuscript titled “FAM50A acts as a novel prognostic marker and modulates the proliferation of colorectal cancer cells via CylinA2/CDK2 pathway”. The authors have investigated FAM50A across different types of cancer and then functionally evaluated the gene in CRC by various experimental methods. The manuscript is well designed and the results are valuable. However, there are several concerns that need to be addressed in this study.

General comments
1.-The title could be changed to “FAM50A as a novel prognostic marker modulates the proliferation of colorectal cancer cells via CylinA2/CDK2 pathway”. The suggested title is shorter and more precise.
2.-What was the rational to evaluate just FAM50A and the authors didn’t investigate FAM50B as well?
3. -There is one article titled “Identification and Clinical Validation of a Novel 4 Gene-Signature with Prognostic Utility in Colorectal Cancer” that pointed to the significance of FAM50A in CRC. So, it could be referenced. Also, the authors could talk about the article in the introduction and discussion.
4. -There are typo/grammar errors that should be revised (i.e. Finally, the changes in the cell cycle following FAM50A knockdown were detected by using flow cytometry; using could be omitted).
ANSWER：

1. Referring to your valuable suggestions, we have revised the title in the article.

2.In our research, the main reason why we focused on FAM50A and didn't conduct an in-depth investigation into FAM50B is that significant differences in expression patterns were found during the detection of tumor tissue samples. The expression level of FAM50A in cancer tissues within tumors is higher than that in adjacent non-cancerous tissues, while the expression level of FAM50B in tumor tissues is lower than that in adjacent non-cancerous tissues. Such an expression characteristic of FAM50A often coincides with the behavioral manifestations of oncogenes. Moreover, our research team has been committed to the field of oncogene-related research for a long time and has a strong interest in and profound research experience regarding molecules with potential oncogene properties. Therefore, we have placed the research focus on FAM50A this time. However, we will also pay attention to the related roles of FAM50B in the future research process.

3. Based on your suggestions, we have read this literature carefully and added a discussion in the article. (Line 105, 107-108, Reference 22)

4. Referring to your suggestions, we have made grammar modifications and checked again.

Abstract
Since the gene expression and prognostic analysis were performed by TCGA and GTEX, its better to mention the databases in the abstract. In the current form it seem that the authors evaluated these variables by experimental analysis in their labs.
ANSWER：Referring to your valuable suggestions, we have mentioned the use of TCGA and GTEX database in the abstract.

Materials and Methods
-The primers sequences should be provided as a table.

ANSWER：Referring to your suggestions, we have already put the primers into the table.

Reviewer #3: The artice is devoted to the actual topic concerning the search of a new prognostic marker for colorectal cancer. The article is clearly written and contains a sufficient amount of introduction and discussion. The results are well structured and illustrated. I recommend the article for acceptance.

同意接收出版。

ANSWER：Thank you very much.
